# Supplementary material for: A study on tuberculosis disease disclosure patterns and its associated factors: Findings from a prospective observational study in Chennai
Source: PLoS One. 2023 Jan 26;18(1):e0280812. doi: 10.1371/journal.pone.0280812 (PMC9879515; doi:10.1371/journal.pone.0280812)
Supplement: Attachment — Submitted filename: REPORT.docx [file pone.0280812.s001.docx]

This paper provides very interesting research work addressing the prospective observational evidence of the TB diseases patterns in Chennai.

1. The introduction section requires substantial improvement. It looks too short and not enough existing studies included. I would suggest adding more literatures and add a conceptual framework.

2. Can you remove the word South India from the title (if possible).?

3. In Table 1, the total frequency has not been included? Can you revise table 1?

4. In Table 2, the total frequency has also not been included? Can you revise table 2?

5. The age-group needs to be reclassified, for example- 0-4 years, 5-14 years, 15-29 years, 30-44 years, 45-59 years, and 60+ years.

6. In Table 5, the adjusted odds ratio for occupation is missing? why? also why the reference category is not arranged properly?

7. The Table 6 is not clear.

8. The title of the Figure 1 is unclear.

9. In Table 4, you have use * sign. But the meaning of * sign is missing in the footnotes.

10. In the result section of the multivariate analysis, the interpretation needs substantial improvement. For-example. include the values of odds ratio with their p value or std.error or confidence interval.

11. What is the strength and drawback of this study?

12.Author should make the integers into one or two decimal places.

13. Any additional policy recommendation?

14. Write the data availability section separately in brief. For example... whether it is publicly available or not. if not then explain why?
