# Supplementary material for: A study on tuberculosis disease disclosure patterns and its associated factors: Findings from a prospective observational study in Chennai
Source: PLoS One. 2023 Jan 26;18(1):e0280812. doi: 10.1371/journal.pone.0280812 (PMC9879515; doi:10.1371/journal.pone.0280812)
Supplement: Attachment — Submitted filename: Response to Reviewers.docx [file pone.0280812.s002.docx]

**Title of the manuscript:** A study on tuberculosis disease disclosure patterns and its associated factors: Findings from a prospective observational study in Chennai

**Manuscript Number:** PONE-D-22-14977

| **S No** | **Reviewer’s Comments** | **Authors responses** | **Page No in Manuscript** |
| --- | --- | --- | --- |
|  | **Reviewer 1** |  |  |
| 1 | Authors should reduce abstract. | As suggested we have edited and reduced abstract to nearly 300 words. | Page -2 |
| 2 | Please state the research questions/hypothesis of the study. | As suggested we have added research question under the method section of the revised manuscript. | Page 5 |
| 3 | Did authors measure internal consistency of their questionnaire, if yes how? | We have used a test -retest method and certain other checks to ensure the internal consistency of the tool which we have used. We have explained this in the method section and have added a published reference which we have made already. | Page 8 |
| 4 | In Statistical analysis, authors should only describe statistical methods, i.e mention statistical techniques/models, and describe why they use in their study.  Please remove outcome and independent variables from the subtitle and make a subtitle, Variable where authors describe about outcome and independent variables separately. | As suggested by reviewer we have revised and separated the statistical methods and outcome/ independent variables under different subtitles. | Page 8 |
| 5 | Authors used univariate and multivariate (multiple) logistic regression model, in multiple logistic regression model consider more than one independent variable, great chance to get multicollinearity problems among selected independent variables, please describe who authors check the multicollinearity problems, and how they overcome the problem if available. | We thank the reviewer for raising this important point. For multiple regressions we calculated variance inflation factor VIA ins STATA and found that all variables had less than 4 and the mean VIA was only 1.63 indicating that that collinearity was not affecting our analysis. We have included this detail in the revised manuscript I results section. | Page 8,12 |
| 6 | Please describe separately inclusion/exclusion criteria, sampling, data collection procedure etc. | As suggested have given separate sub titles and details of inclusion/exclusion criteria, sampling, data collection procedure, analysis, and outcome/ independent variables in the revised manuscript. | Page 5 |
|  | **Reviewer 2 Comments** |  |  |
| 1 | This is an interesting paper based on primary data. The paper is well written . However it need improvement in following | We thank the reviewer for positively acknowledging the importance of our study. We have made the necessary changes as suggested |  |
| 2 | In introduction the social stigma attached to TB in India may be discussed. Second, how the disclosure adversely/ positively affect patients treatment and health may be discussed. | As suggested by the reviewers we have outlined the relationship between disclosure, stigma, poor mental status etc with references in the revised papers. We have added the available literature on positive outcomes of TB disclosure from a setting similar to India. We have also provided a detailed discussion of how disclosures and positive outcomes are important | Page 3,4 |
| 3 | .From title Outcome may be dropped | We have modified the title as suggested by the reviewer | Page 1 |
| 4 | .Tables: All tables should have uniformly 2 decimals. | As suggested we have given 2 decimals uniformly in all tables. | Page 22-27 |
| 5 | Table 3 No. should be replaced with N. Do not show number or % for N<30. there are many cases with N less than 10. Must be replaced with __ mentioning in footnote number not shown due to small number  Put N as last row (all cases) % in parentheses should be shown without bracket It basically shown the percentage of XX by characteristics | As suggested we have replaced the lower numbers . however we have kept it at a level less than <10. This was to highlight incremental changes in disclosures made at different timepoints which are important  We have removed the parenthesis for % in all tables and have made N %as the last row in table header | Page 24 |
| 6 | Keep all column from Odd ratio onwards. Drop the columns except variable | We have dropped the columns before Odds ratio as suggested. | Page 22-27 |
| 7 | A recent paper is missed in literaure review Pathak, D., Vasishtha, G., & Mohanty, S. K. (2021). Association of multidimensional poverty and tuberculosis in India. BMC Public Health, 21(1), 1-12. | As suggested we have added this recent and important literature in the revised manuscript | Page 15 |
|  | **Reviewer 3 Comments** |  |  |
| 1 | This paper provides very interesting research work addressing the prospective observational evidence of the TB diseases patterns in Chennai. | We thank the reviewer for positively acknowledging the importance of our study. |  |
| 2 | The introduction section requires substantial improvement. It looks too short and not enough existing studies included. I would suggest adding more literatures and add a conceptual framework. | As suggested by the reviewers we have improved our introduction section and have added the literature. We have outlined the relationship between disclosure, stigma, poor mental status etc with references in the revised papers. | Page 3-4 |
| 3 | Can you remove the word South India from the title (if possible).? | We have modified the title as suggested by the reviewer | Page 1 |
| 4 | In Table 1, the total frequency has not been included? Can you revise table 1? | We have added the total frequency in Table 1 as suggested | Page 22 |
| 5 | In Table 2, the total frequency has also not been included? Can you revise table 2? | We have added the total frequency in Table 2 as suggested | Page 23 |
| 6 | The age-group needs to be reclassified, for example- 0-4 years, 5-14 years, 15-29 years, 30-44 years, 45-59 years, and 60+ years. | We would like to clarify that the present study included only adult TB patients and their contacts. As such our age categorisation were made from 18 years and above. Our categorisation thus does not have 0-4, 5-14 and 15 plus years. Our categorisation was made based on the prior assumption of TB burden among different age brackets based on TB program data in the study settings. |  |
| 7 | In Table 5, the adjusted odds ratio for occupation is missing? why? also why the reference category is not arranged properly? | Variables which were statistically insignificant IN univariate analysis was not included in multivariate regression. We have added a footnotes to clarify this. Also we have edited the tables and made clearer the reference category and rest. | Page 26 |
| 8 | The Table 6 is not clear. | We have edited the Table 6 title, headers and given footnotes to make it clearer. | Page 27 |
| 9 | The title of the Figure 1 is unclear. | We have edited the title of figure to make it clearer. | Page 28 |
| 10 | In Table 4, you have use * sign. But the meaning of * sign is missing in the footnotes. | We have added the meaning of * sign in the revised manuscript. | Page 25 |
| 11 | In the result section of the multivariate analysis, the interpretation needs substantial improvement. For-example. include the values of odds ratio with their p value or std.error or confidence interval. | As suggested we have added the values of odds ratio, p value and confidence interval and added explanations in the results part. | Page 11 |
| 12 | What is the strength and drawback of this study? | We have added strengths and drawbacks of this study in the discussion part as suggested by the reviewer | Page 16,17 |
| 13 | Author should make the integers into one or two decimal places. | As suggested we have standardised the decimals to two integers | Page 22-27 |
| 14 | Any additional policy recommendation? | We have added policy recommendations in the discussion part as suggested by the reviewer | Page 16 |
| 15 | Write the data availability section separately in brief. For example... whether it is publicly available or not. if not then explain why? | We have added the data availability section as suggested by the reviewer | Page 18 |
